# Professional Secrecy—Dr. Hawthorne's View

**Published:** 1919-06-21

**Authors:** C. O. Hawthorne


### PROFESSIONAL SECRECY—DR. HAWTHORNE'S VIEW.

The writer of your article on "Professional Secrecy" (June 14, p. 258) appears to regret the invasion by legal demand (as, for example, in the Notification of Infectious Diseases Act) of the "honourable tradition" which forbids a physician to reveal to anyone confidences committed to him by his patient. Yet he himself advocates a departure from this tradition in circumstances where the law does not intervene, and where, therefore, any breach of confidence on the part of the practitioner must be entirely free, spontaneous, and voluntary. He is apparently led into these mutually inconsistent attitudes by a contemplation of the position which arises when a person who "is suffering from venereal disease" proposes to marry. His claim is that under these conditions the "honourable tradition" should be ignored, and that any medical practitioner who knows the facts ought to reveal them to the "prospective spouse." Quite naturally the writer dilates on the infamous nature of the intended act, and it is in the prevention of this act that he finds a justification of the sacrifice of the bond of professional secrecy. The argument, on the surface at least, is, it must be admitted, not without its appeal. Yet I will venture to say that it is essentially unsound, that it carries consequences dangerous both to the medical profession and to the public, and that the aim of the policy it proposes can be readily attained without any sacrifice of the "tradition" which, according to your correspondent, may be "more honour'd in the breach than the observance."

The alternative is surely of the simplest, and nothing could be more efficacious. Let every "prospective spouse," either personally or by deputy, demand from the contemplated partner a certificate authoritatively affirming his (or her) freedom from venereal disease—and the thing is done. If the public will not take this plain, straightforward, and obvious course the fault lies with themselves, and such negligence cannot reasonably be urged for the infliction of new responsibilities on the medical profession or for the invasion of a confidential relationship that, on the wide scale, exists in the public interest. Why pay what your writer recognises to be a "high price" for an end that can be obtained without any payment at all? It must be recognised, too, that if a medical practitioner is free on his own initiative to betray his patient's confidence in one respect and for what he believes to be a worthy motive, it will be difficult to call a halt when the same principle is applied in other directions. Once voluntarily abandon the "honourable tradition" and a logical resting-place becomes impossible. Such a demand is particularly ridiculous in circumstances which permit full protection to every person who has the courage and resolution to ask a plain question and to demand a plain answer.

Suppose such a claim allowed, a number of difficult and delicate issues must immediately arise. It is easy to write "suffering from venereal disease," but to define the exact limits of this term will cause much debate. Will early or threatening signs of tabes dorsalis come within the demand? Are the presence of prostatic "threads" in the urine or a positive Wassermann reaction included? These and many other similar questions will have to be settled prior to an agreed interpretation of the proposition that "it is ethically right to reveal to a prospective spouse that the person with whom she contemplates matrimony is suffering from venereal disease." Further, if professional secrecy may be sacrificed to protect intending brides and bridegrooms from venereal disease, why may not the same sacrifice be demanded to afford protection from an epileptic, a tuberculous, or an insane partner, or from one whose family record or personal history shows a decided tendency to one or other of these diseases? If a function of the medical profession is officiously and gratuitously to supervise contemplated or arranged marriages, why halt at venereal disease? Your correspondent appears to think the whole matter one of extreme simplicity and outside the need of argument. Such a conclusion will not be allowed by a profession whose duty it is to respect the confidences of their patients and who must refuse crusades attempted to be imposed on them by well-meaning but uninformed persons. For my own part, I should no more admit the validity of your correspondent's ethics than I should recognise the accuracy of his introductory quotation.

C. O. Hawthorne.

